# Immunophenotypic Profile and Measurable Residual Disease Monitoring in Multiple Myeloma: A Prospective Study From a Tertiary Care Centre in Southern India

**DOI:** 10.7759/cureus.61504

**Published:** 2024-06-01

**Authors:** Pandurangan Sathya, Smita Kayal, Abdoul Hamide, Rakhee Kar

**Affiliations:** 1 Pathology, Jawaharlal Institute of Postgraduate Medical Education and Research (JIPMER), Puducherry, IND; 2 Medical Oncology, Regional Cancer Centre, Jawaharlal Institute of Postgraduate Medical Education and Research (JIPMER), Puducherry, IND; 3 Medicine, Jawaharlal Institute of Postgraduate Medical Education and Research (JIPMER), Puducherry, IND

**Keywords:** survival outcomes, immunophenotyping, measurable residual disease, flow cytometry, multiple myeloma

## Abstract

Background: Multiple myeloma (MM) immunophenotyping (IPT) and measurable residual disease (MRD) monitoring by flow cytometry is a surrogate for progression-free survival and overall survival in clinical trials. However, plasma cell enumeration is challenging owing to morphological discrepancies and plasma cell (PC) loss during the sample processing.

Methods: In (n=87) newly diagnosed MM patients, we evaluated the immunophenotype of PCs at baseline, and for a subset of 35 patients MRD at post-induction was quantified and analyzed for association with outcomes and survival. The software Statistical Package for Social Sciences (SPSS), version 16.0 (SPSS Inc., Chicago, IL, USA) was used for all the statistical analysis.

Results: Immunophenotyping showed strong positive expression of CD56 (83%), CD200 (94%), CD38 (92%), and CD117 (91%) and negative/weak expression of CD19 (83%), CD45 (89%), CD27 (74%), and CD81 (90%) respectively. Negative/weak expression of CD19 was significantly associated with age ≥56 years (p<0.048), with lower albumin (<3.4g/dL, p<0.001). Strong positive CD56 expression was significantly associated with the presence of M-protein (p<0.03). Strong positive CD117 expression was significantly associated with lower albumin (p<0.02). Strong positive CD200 expression was significantly associated with a good response (p<0.02). The median (IQR) value of bone marrow (BM)-MRD% was 0.005 (0.002-0.034). We found that there was no significant difference in the correlation, association, and survival outcomes with MRD%.

Conclusion: This study sheds light on the utility of IPT as an invaluable diagnostic tool in disease management. The findings of this study could be important when it comes to modifying the criteria for high-risk diseases and implementing a risk-adapted first therapy in clinical practice.

## Introduction

Multiple myeloma (MM) is characterized by the abnormal growth of plasma cells (PCs) in the bone marrow (BM), occurring at multiple locations. The presence of monoclonal protein in the blood or urine, as well as evidence of organ damage related to plasma cell disorders (PCD), have been associated with this neoplastic proliferation [1]. The diagnostic criteria include the presence of >10% clonal PCs or biopsy-proven bone or extramedullary plasmacytoma and any one or more of the myeloma-defining events (MDE) as established by the International Myeloma Working Group (IMWG) [2]. Although various newer treatments, including immune modulatory drugs and proteasome inhibitors, succeeded by Autologous Stem Cell Transplantation (ASCT), have proven effective in individuals with myeloma, [3]; MM being a heterogenous disease with varied clinical outcomes it is important to prognosticate patients [4]. Although the clinical outcomes of MM patients have improved, some patients continue to show resistance to the aforementioned therapy. Response to treatment can be evaluated by flow cytometry-based measurable residual disease (MRD) which is a well-known predictor of progression-free survival (PFS) and overall survival (OS) among patients [5].

Multiparametric flow cytometry (MFC) has been a powerful technique for analyzing cell surface and intracytoplasmic markers. Due to its high sensitivity, specificity, and rapid results, it has become a cornerstone in disease diagnosis and differentiation. In recent years, MFC has become increasingly significant for the diagnosis, monitoring, and prognosis of PCDs, particularly MM [6]. MFC holds clear clinical relevance in various aspects of clonal PC disorders: distinguishing between MM and other PC-related disorders in the differential diagnosis [7]. Flow cytometry has proven valuable in distinguishing neoplastic PCs (neoPCs) from reactive PCs (rPCs) through analysis of their distinct surface antigen expressions and clonality [8].

Immunophenotyping (IPT) is widely used for the diagnosis of a variety of hematologic malignancies, including MM, as well as the evaluation of measurable residual illness and the prediction of patient prognosis. Neoplastic PCs typically exhibit under-expression of CD19, CD45, CD27, CD81, and CD38, while showing over-expression of CD56, CD117, CD28, CD33, and CD200. In contrast, reactive PCs predominantly express CD19, CD45, CD27, and CD81, while lacking CD56 expression. However, there may be minor populations within reactive PCs that express surface antigens differently [7,8]. Moreover, unlike normal PC, myeloma cells commonly lack both the mature plasma cell antigen and the leukocyte common antigen (CD45) [7]. There are discrepancies in the exact immunophenotype of normal and abnormal plasma cells and their association with disease prognosis [8].

The integration of MFC has expanded the immunophenotypic analysis of MM. MFC allows a more comprehensive assessment of surface markers and facilitates the identification of MRD with enhanced sensitivity. MRD assessment is crucial for monitoring treatment response and predicting patient outcomes, ultimately guiding clinical decisions [9]. It offers prognostic insights, predicting the risk of progression in smoldering MM (SMM) and identifying active MM patients with unfavorable outcomes or unexpected long-term survival despite incomplete responses. MFC can also characterize monoclonal gammopathy of undetermined significance (MGUS)-like phenotypes [10].

The measurement and achievement of MRD-negative BM in MM patients, as determined equally by MFC or next-generation sequencing (NGS) methods, is becoming more clinically relevant [11]. MRD refers to the presence of myeloma cells in the bone marrow after a clinical response has been detected and the patient is in morphologic remission [11]. These remaining myeloma cells are clinically significant because they have the potential to cause disease progression and recurrence. Due to advancements in MM therapy, the response criteria have changed over time. A typical complete response (CR) requires bone marrow aspirates with less than 5% plasma cells, regardless of clonality. The expression of cell-surface markers and genomic architecture can now be used to further identify myeloma cells. Although previous research has indicated that MRD might serve as a promising biomarker for assessing treatment effectiveness, aiding in therapeutic decision-making, and acting as a surrogate for overall survival, the significance of MRD in MM remains a subject of debate [9].

Hence, this study was done to assess the bone marrow immunophenotypic profile at baseline, quantify residual disease after treatment by flow cytometry in MM, and check their association with other baseline parameters, response, and survival outcomes.

## Materials and methods

Study design and setting

This was a prospective, cross-sectional analytical study conducted over three and a half years, from January 2018 to July 2021. It was carried out in the Department of Pathology in collaboration with the Departments of Medical Oncology and Medicine at Jawaharlal Institute of Postgraduate Medical Education and Research (JIPMER), Puducherry. Approval for this study was obtained from the Institutional Ethics Committee, JIPMER (JIP/IEC/2017/0146).

Study subjects

All newly diagnosed MM patients undergoing routine diagnostic workups were included in the study after obtaining written informed consent and a convenient sampling was performed. MM was diagnosed using the most recent WHO criteria [12]. The immunophenotypic profile was determined using 8-10 color flow cytometry on bone marrow aspirate (BMA) samples for diagnosis. After completion of induction therapy, the level of MRD% was assessed on BMA wherever feasible.

Sample collection

As part of a routine diagnostic workup, BMA samples from the iliac crest were obtained in an anticoagulant (ethylenediaminetetraacetic acid or EDTA) stabilized medium, following standard protocol, and all aseptic precautions. The study involved no additional invasive procedure. For immunophenotyping and measurable residual disease analysis, the BMA sample was collected in the same syringe immediately after obtaining the first 0.2 to 0.5 ml sample for slide morphology, ensuring no dilution of morphology. Within 24 hours of sample collection, processing, and data acquisition were completed.

Sample processing

Lysing and Washing

All the reagents and consumables were procured from Beckman Coulter Life Sciences, USA. The lyse-wash-stain method was used for sample processing at the time of diagnosis and after treatment. Using ammonium chloride-based lysing reagent (0.15 M NH4Cl (8.29 g NH4Cl), 1.0 g KHCO3, 37 mg EDTA, and 1 L distilled water) erythrocytes were bulk lysed to prepare the cell suspension. The total leucocyte count was calculated from an automated hematology analyzer to get the final concentration of 1 to 2 × 106 cells/µl and the sample was diluted appropriately if it was >50,000 cells/cu.mm. The pre-titrated volume of the monoclonal antibodies was used to stain the cells. For immunophenotyping, an in-house antibody cocktail and DuraClone RE-PC (B80394-25) panels were used. In-house prepared antibody cocktails were used to assess the level of MRD% (Table 1).

**Table 1:** Panel of antibodies and their fluorochromes utilized for MM characterization and MRD assessment. MRD, Measurable Residual Disease; IPT, Immunophenotyping; RE-PC, Rare events plasma cell tube; FITC, Fluorescein isothiocyanate; PE, Phycoerythrin; ECD: Energy-coupled dye; PC5.5, Phycocyanin; PC7, Phycocyanin 7; APC, Allophycocyanin; APC700, Allophycocyanin 700; APC750, Allophycocyanin 750; PB, Pacific blue; KrO, Chrome orange.

Fluorochrome	FITC	PE	ECD	PC5.5	PC7	APC	APC 700	APC 750	PB	KrO
											IPT-Tube-1	CyIgκ	CyIgλ	×	CD138	CD19	CD56	CD200	CD38	CD27	CD45
IPT-Tube-2/MRD-Tube-2	CD81	CD117	×	CD138	CD19	×	CD200	CD38	×	CD45											
DuraClone RE-PC panel	CD81	CD27	×	CD19	CD200	CD138	×	CD56	CD38	CD45

Staining

Staining was done with the fluorochrome-tagged surface or intracellular antibodies CD19, CD45, CD38, CD138, CD56, CD117, CD27, CD81, CD200, and cytoplasmic immunoglobulin light chain kappa and lambda. Initially, the staining with a pre-titrated volume of the surface antibodies was performed with 100 µl of lysed cells used in all cases. The mixture was vortexed and left in the dark for 20 minutes. The washing of excess unbound antibodies by adding 2 ml of phosphate-buffered saline (PBS) was performed, vortexing, centrifuging for five minutes at 2,500 revolutions per minute (RPM), and discarding the supernatant.

The intracytoplasmic antibodies were fixed with formaldehyde and permeabilized with saponin (Intraprep R1 and R2^TM^). This step was followed by adding intracytoplasmic antibodies, incubation, and washing. An unstained tube was processed along with each panel to check for autofluorescence. The diluent (Iso Sheath Fluid^TM^) was used for cell suspension and acquisition. Finally, the instrument’s undesired debris was removed by proteolytic enzyme solution (Cleanze^TM^).

Sample acquisition and analysis

Sample acquisition was performed using a flow cytometer (Navios-AY43297, Beckman Coulter, USA) equipped with a 3-laser (10-color) system. A range from a minimum of 0.1 million events up to a maximum of 3.0 million events was acquired. When leucocyte counts were low, we tried to acquire a greater number of events in such instances. The data were analyzed using Kaluza version 2.1 software (Beckman Coulter, USA) to distinguish between reactive PCs and neoplastic plasma cells and assess for the MRD%. Residual myeloma cells identified by myeloma-associated immunophenotype (MAIP) which was based on either aberrant antigen expression or antigen loss/under-expression or over-expression were taken as the numerator; viable nucleated cells after exclusion of debris formed the denominator using the forward scatter (FSC) versus side scatter (SSC) gate.

Calculation of MRD%

The formula for calculating MRD%: The number of MRD events/the total number of viable events or nucleated events×100.

Lower Limit of Detection (LLOD) and Lower Limit of Quantification (LLOQ)

LLOD was used as 30 events/total number of viable cells analyzed×100. Similarly, the LLOQ was estimated as 50 events/total number of viable events analyzed×100. Thus, the LLOD and LLOQ were both typically dependent on the total number of events analyzed.

Quality Measures for the Flow Cytometer

Cytometer stability and sensitivity were verified daily, and quality control measures were implemented using Cytometer Setup and Tracking (CS&T) beads according to the manufacturer’s instructions.

Gating Strategy and Analysis of Immunophenotyping and Residual Disease Monitoring

The strategy of sequential gating was used. An FSC versus time plot was created to verify the instrument’s fluidics and acquisition time. A wide singlet gate was used to include larger and binucleated plasma cells, based on the FSC-peak versus FSC-area. A broad "scatter-gate" was plotted to exclude dead cells and debris, against SSC versus FSC. Lymphocytes were selectively gated by plotting SSC versus CD45 to eliminate normal T-lymphocytes which typically express CD38. A "liver-quadrant" gate was designated as CD38+ve events were plotted alongside the CD38 versus CD45 plot, which showed CD38 bright positive and CD45 dim to negative. The dual expression of plasma cells was plotted using CD38 and CD138. These PCs were further analyzed for the presence and absence of other markers in the cocktail to distinguish between the reactive and neoplastic plasma cells, which were subsequently confirmed based on light chain expression. Boolean gates were created for neoplastic plasma cells, reactive plasma cells, and lymphocytes using the 'OR' Boolean operator for subsequent analysis. A hinged gate was employed for kappa and lambda plots. The cytoplasmic immunoglobulin light chain restriction of these cells is confirmed by clonality.

Data Obtained From the Patient’s Medical Records

The patient’s medical records were reviewed for demographic, clinical, and laboratory information including age, gender, comorbidities like diabetes mellitus and hypertension, renal failure, bone lesions, and eastern cooperative oncology group performance status (ECOG-PS). Other laboratory information includes routine biochemical tests, serum-free light chain (sFLC), serum protein electrophoresis/immunofixation findings and hematological findings, staging, therapeutic details (induction regimen), and outcomes (post-induction and survival data). Wherever reports of fluorescence in situ hybridization (FISH) were available, they were collected and analyzed. Based on the IMWG response criteria, a response assessment was done. The date of start of treatment, duration of time till the disease progressed or died, and the last follow-up date were all recorded. The period from the beginning of treatment until the incidence of progression or death from any cause was called progression-free survival (PFS). Overall survival (OS) was defined as the time from the initiation of treatment until death from any cause.

Statistical analysis

Statistical analysis was done using the software Statistical Package for Social Sciences (SPSS), version 16.0 (SPSS Inc., Chicago, IL, USA). The Kolmogorov-Simonov test was used to assess the normality of the data for continuous variables. Continuous variables were summarized using the mean ± SD or median (IQR) based on the data distribution, while categorical data were presented as frequency and percentages. A comparison of categorical data among the two variables was done using the chi-squared test. The Spearman’s test was used to analyze the correlation between the two variables. The impact of baseline characteristics on survival was studied using the Cox proportional hazard model. For association studies, treatment response has been taken as good (VGPR {very good partial response} and above) and poor (PR {partial response} and below). The Kaplan-Maier method along with the log‐rank test, was used to analyze the survival outcomes (PFS and OS) and assess response. A P-value less than 0.05 was considered statistically significant.

## Results

In the current study, newly diagnosed MM patients (n=87) at baseline and MM patients at response assessment (n=35) were prospectively enrolled and MFC analysis was performed.

Baseline characteristics of study participants

The patient and disease characteristics of these (n=87) consecutive newly diagnosed MM patients are given in Table 2. The median age of the patients was 55 years with a higher proportion of males 55 (63%) and the majority of the patients 53 (65%) were in International Staging System (ISS) stage III disease. An ECOG-PS ≤ 2 was present in 60 (69%) of the patients. The median (IQR) levels of lactate dehydrogenase (LDH), serum albumin, and β2-microglobulin (β2-µg) were 232.50 (179.75-473.20) (units/L), 3.4 (2.9-4.0) (g/dL), and 5999.50 (3579.25-14965.50) (ng/mL) respectively. The presence of serum M-protein, BJ-protein, and bone lesions in the study group was 69 (81%), 33 (51%), and 74 (91%) respectively. FISH studies for cytogenetic abnormalities showed positive abnormalities in 13 (46%), whereas 19 (54%) of the patients were found to be normal. At our hospital, a combination of proteasome inhibitors, immunomodulatory agents, and steroids is the recommended standard of treatment for MM induction therapy. The selection of a drug combination is determined by the presence of comorbidities, the degree of renal dysfunction, and the patient's overall fitness status. The median number of chemotherapy cycles administered was 6, with a range of 4 to 8. Bortezomib+Lenalidomide+Dexamethasone (VRd), Bortezomib+Dexamethasone (Vd), and Bortezomib+Pomalidomide+Dexamethasone (VPd) were the most frequently used induction regimens for the patients, accounting for 31 (40%), 18 (23%), and 13 (17%) of the patients, respectively. After a patient reached VGPR and above, 45 (66%) underwent ASCT transplantation.

**Table 2:** Baseline characteristics. *Continuous variables are expressed as Mean ± SD, Median (IQR); # Categorical variables are expressed as frequency and percentage. MM, Multiple myeloma; IQR, Interquartile range; SD, Standard deviation; n, Number; %, Percentage; LDH, Lactate dehydrogenase; BJ-protein, Bence-Jones protein; ISS, International Staging System; ECOG-PS, Eastern Cooperative Oncology Group Performance Status; VRd, Bortezomib+Lenalidomide+Dexamethasone; CyBorD, Bortezomib+Cyclophosphamide+Dexamethasone; Vd, Bortezomib+Dexamethasone; VCd, Bortezomib+Cyclophosphamide+Dexamethasone; VPd, Bortezomib+Pomalidomide+Dexamethasone; VTd, Bortezomib+Thalidomide+Dexamethasone; DCEP, Dexamethasone+Cyclophosphamide+Etoposide+Cisplatin.

S. No.	Variables	MM patients (n=87) Median (IQR), n (%)
Demographic features of the study participants (n=87)		
1	Age in years*	55 (48-63)
2	Gender	
	Male	55 (63)
	Female	32 (37)
Laboratory parameters of the study participants (n=87)*		
3	Hemoglobin (g/dL)	8.2 (6.8-10.7)
4	Serum calcium (mg/dL)	9.9 (8.4-11.1)
5	Serum creatinine (mg/dL)	1.26 (0.78-3.16)
6	LDH (units/L)	232 (179-473)
7	Serum albumin (g/dL)	3.4 (2.9-4)
8	Beta-2 microglobulin (ng/mL)	5999 (3579-14965)
9	Serum M-protein (g/dL)	2.81 (1.27-4.80)
10	Serum M-protein (n= 85)	
	Present	69 (81)
	Absent	16 (19)
Clinical characteristics of the study participants^ #^		
11	BJ-protein (n=65)	
	Present	33 (51)
	Absent	32 (49)
12	Bone lesions (n=81)	
	Present	74 (91)
	Absent	7 (9%)
13	ISS staging (n=82)	
	ISS-I & ISS-II	29 (35)
	ISS-III	53 (65)
14	ECOG-PS (n=87)	
	0-2	60 (69)
	3-4	27 (31)
15	Induction regimen (n=74)	
	VRd	31 (40)
	CyBorD	3 (4)
	Vd	18 (23)
	VCd	1 (1)
	VPd	13 (17)
	VTd	6 (8)
	DCEP	2 (3)
16	Transplantation status (n=68)	
	Eligible	45 (66)
	Ineligible	23 (34)

Correlation of bone marrow FCM-PC% with other laboratory parameters

The median level of plasma cells in bone marrow by flow cytometry (FCM-PC%) was 4.94 (1.37-18.41), while by morphology (BM-PCs%) it was 50, with a range between 25 to 75. The correlation between FCM-PC% with demographic and laboratory parameters is shown in Table 3. There was a significant weak positive correlation between FCM-PC% and β2-µg (p<0.02); serum creatinine (p<0.02), moderate positive correlation with BM-PC% (p<0.0001), and weak negative correlation with hemoglobin (Hb) (p<0.01), respectively.

**Table 3:** Correlation between demographic and laboratory parameters with FCM-PC%. LDH-lactate dehydrogenase; FCM-PC, flow cytometry plasma cell; BM-PC, bone marrow plasma cell; r, Coefficient of correlation; β2-µg, Beta-2 microglobulin; p<0.05 statistical significance; n, Number; %, Percentage.

S. No.	Parameters	r-value	p-value
1	Age in years (n=87)	0.03	0.72
2	LDH (n=58)	0.01	0.89
3	Serum albumin (n=87)	-0.01	0.87
4	β_2_-µg (n=70)	0.26	0.02
5	Hemoglobin (n=87)	-0.27	0.01
6	Serum calcium (n=87)	0.12	0.27
7	Serum creatinine (n=66)	0.23	0.02
8	Serum M-protein (n=66)	0.2	0.09
9	BM-PC (%) (n=85)	0.49	0.0001

Expression pattern of cluster of differentiation (CD) markers and their intensity

The intensity of the expression pattern of CD markers in neoplastic PCs is shown in Table 4. Immunophenotyping showed strong positive expression in CD56 (83%), CD200 (94%), CD38 (92%), and CD117 (91%) and negative/weak expression of CD19 (83%), CD45 (89%), CD27 (74%), and CD81 (90%) respectively.

**Table 4:** Expression pattern of CD markers and their intensity. CD, Cluster of differentiation; n, Number; %, Percentage.

S. No.	CD markers	Total	Neoplastic plasma cells (n=87), n (%)	
			Negative/weak	Moderate/strong/variable
					1	CD19	87	72 (83)	15 (17)
2	CD45	87	78 (89)	9 (11)
3	CD56	87	15 (17)	72 (83)
4	CD27	39	29 (74)	10 (26)
5	CD81	39	35 (90)	4 (10)
6	CD200	36	2 (6)	34 (94)
7	CD117	36	24 (42)	12 (58)
8	CD38	87	7 (8)	80 (92)
9	CD138	85	4 (5)	81 (95)

**Figure 1:**
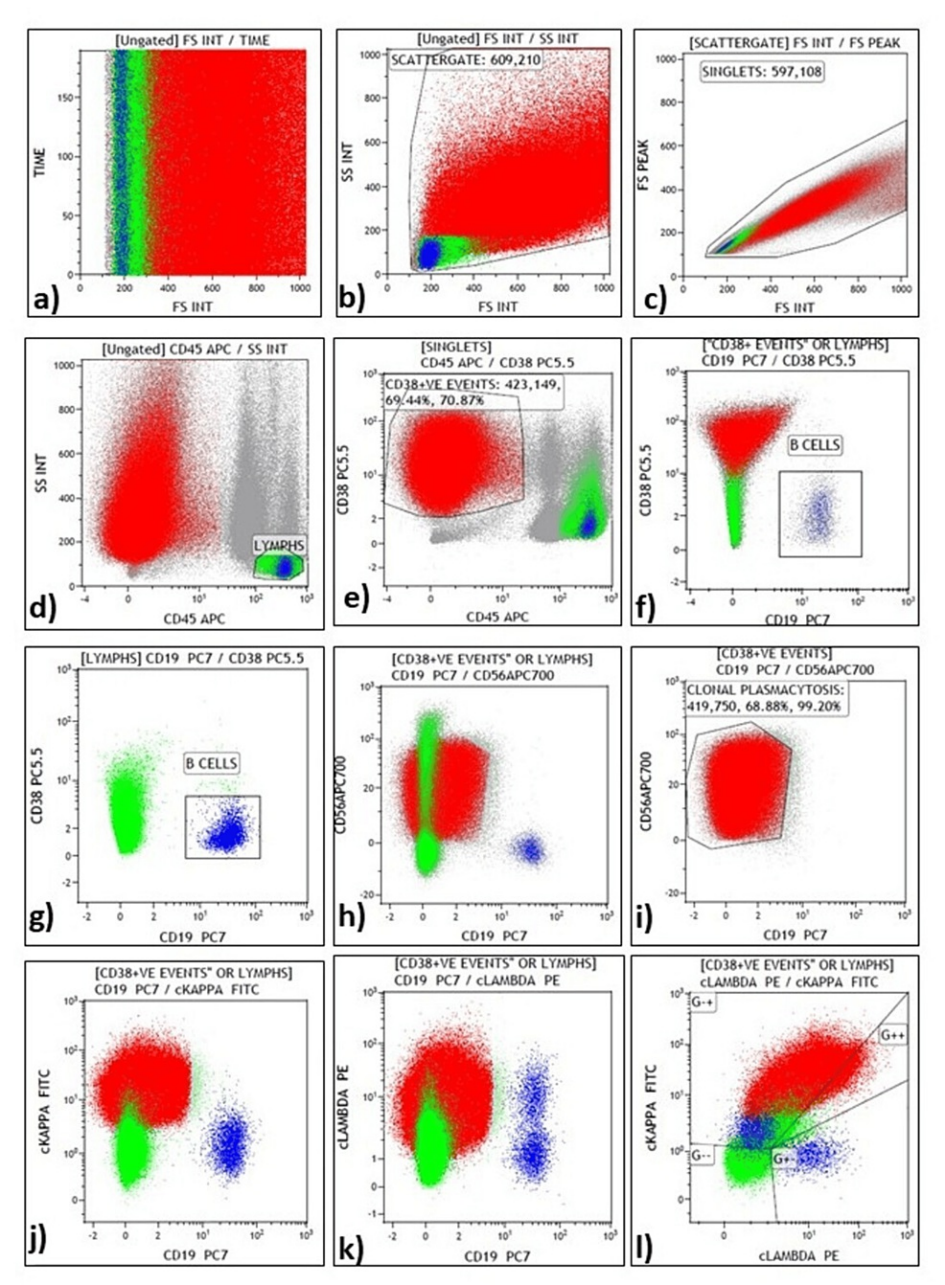
Bone marrow immunophenotyping by flow cytometry in a case of newly diagnosed MM. (a) Time versus forward scatter (FSC) demonstrating the acquisition integrity; (b) A scatter gate based on side scatter (SSC) versus FSC was used to eliminate debris; (c) Wide singlet gate was applied to the FSC peak versus FSC integral plot; (d) Lymphocytes were gated on SSC versus CD45; (e) CD38 versus CD45 was used to gate CD38 bright positive events forming a distinct cluster in CD45 negative region; (f) CD38 positive events and lymphocytes in CD38 versus CD19 showing these plasma cell cluster to be negative for CD19; (g) Lymphocytes were seen in CD38 versus CD19 plot with gated B-cells; (h, i) CD38 positive events and lymphocytes and the former alone visualized in CD56 versus CD19 plot showing strong expression of CD56 in plasma cells (PCs); (j-l) Kappa and lambda plots showing kappa restriction in the neoplastic PCs and polyclonal expression in B-lymphocytes. There are hardly any reactive plasma cells in this sample. MM, Multiple myeloma.

**Figure 2:**
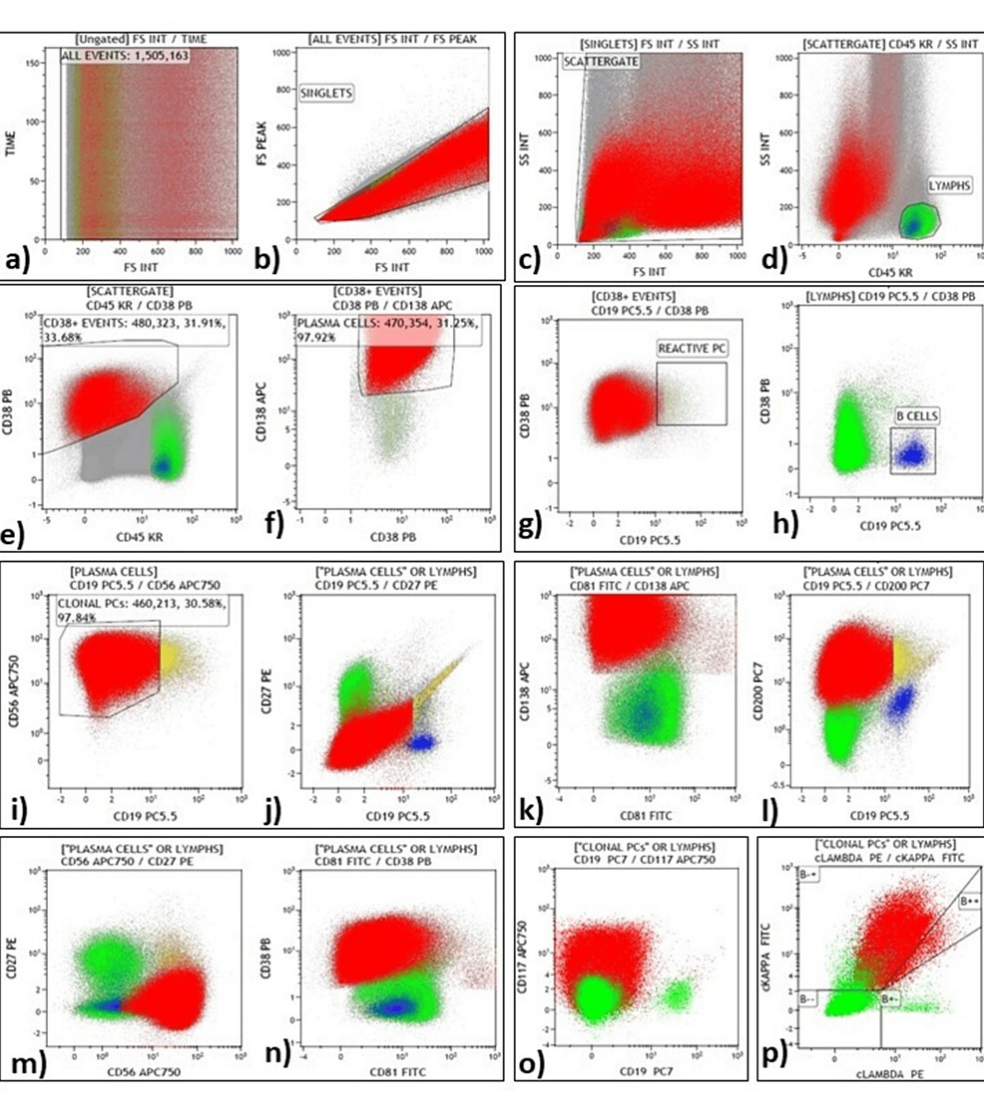
Bone marrow immunophenotyping by flow cytometry in a case of newly diagnosed MM using DuraClone RE-PC tube. (a) Time versus forward scatter (FSC) demonstrating the acquisition integrity; (b) A wide singlets gate was applied to the FSC peak with an FSC integral plot; (c) A scatter gate based on side scatter (SSC) versus FSC was used to eliminate debris; (d) Lymphocytes were gated on SSC versus CD45 plot; (e) In the CD38 versus CD45 analysis, gating was applied to CD38 bright positive events than lymphocyte positivity; (f) CD38 positive events seen in CD138 versus CD38 plot, showed dual positivity gated as plasma cells (PCs); (g) In CD38 versus CD19 plot, CD19 positive events were gated as reactive PCs; (h) Lymphocytes seen in CD38 versus CD19 plot, CD19+ve events were gated as B-lymphocytes; (i) PC events were visualized in CD56 versus CD19 plot, CD19 negative cluster gated as clonal PCs; (j-o) Boolean gates of neoplastic PCs showing under expression of CD27 and CD81, strong expression of CD200, CD56 and moderate expression of CD117; (p) cytoplasmic Kappa and lambda graph showing kappa restriction. MM, Multiple myeloma; RE-PC, Rare events plasma cell.

The analysis strategy of BM-IPT and representative plot for gating in MM cases are shown in Figure 1 and Figure 2.

Association of baseline immunophenotypic CD markers with demographic, laboratory, and clinical characteristics

The relationship between clinico-demographic characteristics and laboratory parameters with expression patterns of immunophenotypic markers was studied. For the continuous variables like age, serum albumin, creatinine, BM-PC%, FCM-PC%, etc. the median value was taken as a cut-off for the association studies. Categorical variables like gender, presence or absence of M-protein, etc. were compared for the association with the expression pattern of immunophenotypic markers.

Negative/weak expression of CD19 was significantly associated with age ≥55 years (p<0.048) with lower albumin (<3.4g/dL, p<0.001), a higher BM-PC% (p<0.002), and higher FCM-PC% (p<0.002) and relapse (p<0.009). Strong positive expression of CD56 was significantly associated with the presence of M-protein (p<0.03) and higher FCM-PC% (p<0.01). Strong positive expression of CD200 was significantly associated with a good response (p<0.02). Strong positive CD117 expression was significantly associated with lower albumin (p<0.02) and higher BM-PC% (p<0.01). A strong positive for CD38 was significantly associated with good response and survival at the last follow-up. Since CD138 expression was taken as a specific gating marker for plasma cells most of the cases show strong positive expression. Hence, it was not used for the association studies.

Factors affecting survival outcomes: univariate and multivariate analysis

The survival outcome for different variables for this subset of patients was analyzed. Based on the median values of FCM-PC%, the data was categorized into two groups ≥4.94 and <4.94. However, no significant association was seen between FCM-PC% and median PFS and OS. When other variables were compared with survival outcome, poor response and higher stage hazard ratio (HR=4.97, p<0.001 and HR=2.62, p<0.02, respectively) were associated with worse OS. Additionally, the findings of multivariate analysis also revealed that a poor response outcome exhibited a higher hazard ratio (HR=4.93, p<0.02) and worse OS. Table 5 shows the survival outcome and its association with FCM-PC% and other variables.

**Table 5:** Factors predicting worse progression-free survival and overall survival using univariate analysis. FCM-PC, Flow cytometry plasma cell, BJ-protein, Bence-Jones protein; FISH, Fluorescence in situ hybridization; Hb-hemoglobin; LDH, lactate dehydrogenase; M-protein, monoclonal protein; β2-µg, Beta-2 microglobulin; ISS, International Staging System; ECOG-PS, European Cooperative Oncology Group Performance Status; PFS, Progression-free survival; OS, Overall survival; n-Number; %, Percentage; p<0.05 statistical significance; NR, Not reached.

Variables	Total	Median PFS (months)	95% (confidence interval)	Hazard ratio	p-value	Median OS (months)	95% (confidence interval)	Hazard ratio	p-value
FCM-PC%									
<4.94	44	31	17.61 - 44.38	1	0.77	35	14.87 - 55.12	1	0.98
≥4.94	43	39	28.05 - 49.94	1.13		NR	19.63 - 32.76	1.00	
Age									
<55 years	44	30	10.20 - 49.79	2.01	0.08	NR	26.80 - 46.53	1.44	0.29
≥55 years	43	39	21.23 - 56.76	1		25	20.97 - 38.54	1	
Gender									
Male	55	30	22.51 - 37.48	1.60	0.31	NR	25.83 - 41.13	1.39	0.34
Female	32	39	20.21 - 57.78	1		25	1.11 - 57.84	1	
ECOG-PS									
ECOG 0-2	60	39	32.79 - 45.20	1	0.08	42	18.17 - 65.82	1	0.15
ECOG 3-4	27	19	3.50-34.49	2.05		11	1.70 - 20.29	1.66	
ISS staging									
I & II	29	40	23.02 - 56.97	1	0.44	NR	32.40 - 52.93	1	0.02
III	53	31	22.76 - 39.23	1.37		14	1.11 - 35.12	2.62	
BJ-protein									
Absent	32	25	6.95 - 43.04	1	0.46	25	20.96 - 52.71	1	0.92
Present	33	34	27.80 - 40.19	1.45		35	2.91 - 67.08	1.04	
LDH									
<232.50	29	40	30.99 - 49.00	1	0.78	NR	22.09 - 36.33	1	0.06
≥232.50	29	39	14.68 - 63.31	1.17		9	1.11 - 25.80	2.19	
Albumin									
<3.4	46	26	23.74 - 28.25	1.40	0.41	25	21.03 - 40.71	1.64	0.15
≥3.4	41	39	30.55 - 47.44	1		42	27.90 - 56.09	1	
β2-µg									
<5999.50	35	39	23.75 - 54.24	1	0.37	42	15.48 - 68.51	1	0.86
≥5999.50	35	31	9.59 - 52.41	1.49		35	21.73 - 40.45	1.06	
Response									
Good	35	39	23.96 - 54.03	1	0.19	42	32.07 - 51.92	1	0.001
Bad	17	12	1.11 - 44.77	1.83		9	5.25 - 12.74	4.97	
Serum calcium									
≥9.9	42	31	13.75 - 48.24	1.23	0.63	25	2.70 - 47.29	1.58	0.19
<9.9	45	34	19.83 - 48.16	1		NR	29.82-48.19	1	
Serum creatinine									
<1.26	45	39	27.76 - 50.23	1	0.45	42	18.30 - 65.69	1	0.10
≥1.26	42	34	18.28 - 49.71	1.42		9	19.09 - 37.87	1.74	
Hb									
≥8.2	41	39	24.74 - 53.25	1.07	0.86	42	12.57 - 71.43	1.90	0.06
<8.2	46	31	24.91 - 37.08	1		35	1.11 - 72.95	1	
M-protein									
Absent	16	34	6.20 - 61.79	1	0.79	NR	19.42 - 33.62	1	0.82
Present	69	31	18.63 - 43.36	1.15		42	17.11 - 66.88	1.45	
Bone lesions									
Absent	7	NR	26.16 - 44.83	1	0.18	NR	16.09 - 42.81	1	0.51
Present	74	34	25.68 - 42.31	3.55		35	12.32 - 57.67	1.60	
FISH									
Negative	11	11	5.22 - 16.72	1	0.05	NR	18.49 - 54.08	1	0.96
Positive	15	NR	25.04 - 42.29	4.35		NR	16.72 - 36.13	1.03	

Level of BM-MRD% and proportion of positive and negative cases

**Figure 3:**
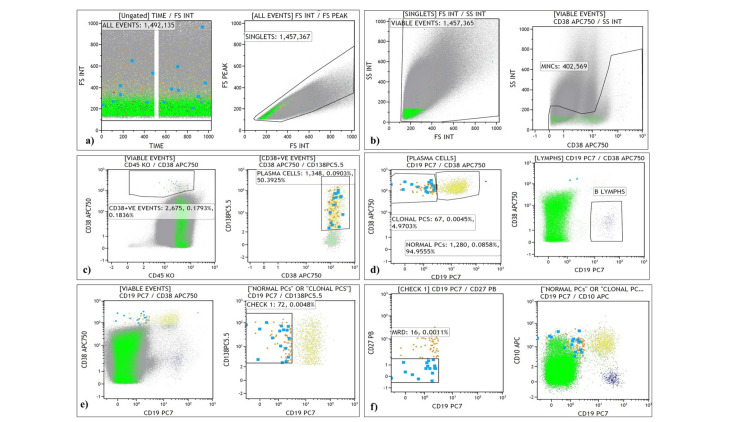
Flow cytometry gating approach for MRD measurement using BMA sample at post-induction in MM. (a-b) Plots showing sample acquisition and integrity, and gating of CD38 positive mononuclear cell (MNC) population, (c-e) Gating of plasma cells (PCs) and demarcation of clonal and normal PCs, (f) CD27 under expressing cells among neoplastic PCs gated as MRD. MRD, Measurable residual disease, BMA, Bone marrow aspirate; MM, Multiple myeloma.

**Figure 4:**
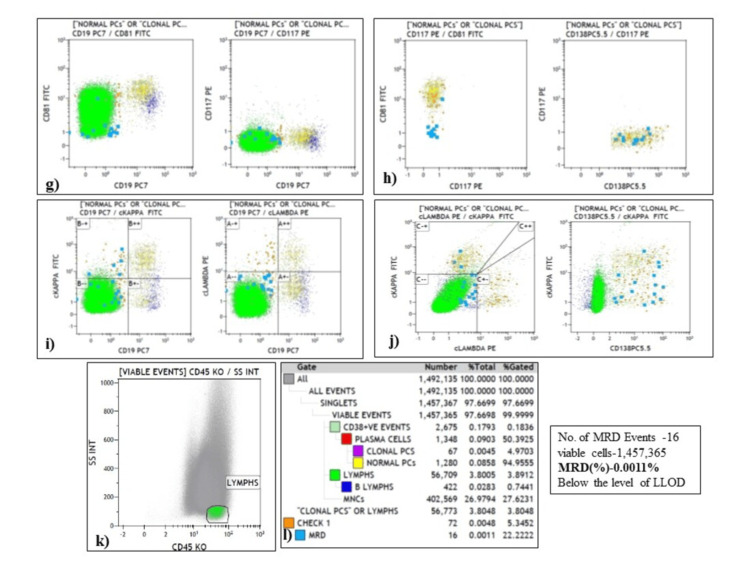
Flow cytometry gating approach for MRD measurement using BMA sample at post-induction in MM. (g, h) MRD events are negative for CD117 and show under expression of CD81 concerning normal plasma cells; (i-k) The clonality was assessed using cytoplasmic kappa and lambda plot, MRD events showing kappa restriction; (l) Number and percentage of gated events followed by calculation of MRD% was showed in hierarchical box plot. MRD, Measurable residual disease, BMA, Bone marrow aspirate; MM, Multiple myeloma.

To determine BM-MRD, a standard cut-off level is ≥0.01% for acute leukemia. The median (IQR) value of BM-MRD% for our subset (n=35) of MM patients was 0.005 (0.002-0.034). However, there are not many studies were done on MRD in MM patients. Hence, BM-MRD% was divided into two categories, positive (≥0.005) and negative (<0.005) based on the median value. MRD was found to be negative in 23 (66%) patients with a median (IQR) value of 0.003 (0.001-0.006) and positive in 12 (34%) patients with a median (IQR) value of 0.10 (0.023-1.725). The analysis strategy of MRD and representative plot for gating is shown in Figure 3 and Figure 4.

Correlation and association of BM-MRD% level with other clinical parameters and survival outcome

On correlation studies, the relationship between BM-MRD% with Hb (r=-0.12, p=0.47), serum albumin (r=-0.14, p=0.42), β2-µg (r=0.10, p=0.61), serum creatinine (r=-0.04, p=0.82), and LDH (r=-0.02, p=0.89) as well as BM-PC% at post-induction by morphology (r=0.11, p=0.53) was not statistically significant.

In association studies, similar BM-MRD% with gender (p=0.91), ISS-staging (0.99), ECOG-PS (p=0.17), cytogenetic abnormalities (p=0.19), BJ-protein (p=0.43), and response outcome (p=0.42), did not show any significant association.

There was no significant difference in the survival outcomes of patients with MM between those with MRD-positive and MRD-negative.

## Discussion

Immunophenotyping appears to be an essential tool in the treatment of hematological malignancies and plays an important role in the diagnosis, prognosis, and treatment of plasma cell diseases. MFC has developed significantly, to screen more events and simultaneously identify more antigens. Specific CD marker expression patterns can be used to characterize the disease and predict treatment response outcomes. In our study, we performed bone marrow immunophenotyping in 87 MM patients at baseline 35 at response assessment analysed its association with baseline characteristics and prognostic indicators, and studied response and survival outcomes with the immunophenotypic characteristics.

Our findings about the demographic characteristics in this study were compatible with those from previous MM studies carried out in Sub-Saharan Africa, there is a predominance of males and a median age at presentation ranging from 53 to 62 years [13]. Compared to studies done in primarily Caucasian populations, such as Kyle et al. and Kristinsson et al., which showed a median age of 66 and 70 years, respectively [14,15]. In contrast, the median age of our cohort of patients was 55 years and hence younger by almost a decade at diagnosis.

The study assessed the expression of various CD markers as prognostic markers and these results showed predominantly negative expression for the following markers CD19, CD45, CD27, and CD81. In contrast, CD138, CD38, CD56, CD200, and CD117 exhibited moderate to strong positive expression. Significant associations were found between certain CD markers and clinical parameters. Negative/weak expression of CD19 was significantly associated with age ≥56 years, lower albumin (<3.4g/dL), a higher BM-PC% and FCM-PC%, and relapse. Strong CD56 positive expression was significantly associated with the presence of M-protein and higher FCM-PC%. Strong CD200 positive expression was significantly associated with a good response. Strong positive CD117 expression was significantly associated with lower serum albumin and higher BM-PC%. A strong positive for CD38 was associated with good response and survival at the last follow-up.

In previous literature, several studies have investigated the role of IPT in MM. These studies have explored different CD markers and their associations with disease characteristics, treatment response, and survival outcomes. Some common markers that have been studied include CD19, CD45, CD27, CD56, CD38, CD138, CD81, CD117, and cytoplasmic kappa and lambda among others [14]. In a study conducted by Guo et al., among 79 newly diagnosed MM patients, CD117-, CD28+, and CD45+ were significantly associated with shortened PFS and poor ISS staging [16]. In another study, MM patients treated with bortezomib and dexamethasone CD45-ve expression were significantly associated with shortened time to the next therapy and PFS. However, CD56 and MPC-1 expression were not associated with survival outcomes [17]. Whereas in another study conducted by Bataille et al., CD56- expression was associated with reduced OS compared to CD56+ expression [18]. The expression of CD19, CD28, and CD117 was significantly associated with shortened PFS and OS. Simultaneous assessment of CD19, CD28, and CD117 classified patients into three different groups namely: poor risk (CD28+ and CD117-), intermediate risk (either negative or positive), and good risk (CD28-and CD117+) [19]. In another study, MFC scoring was done based on the positive expression of CD56 and CD20 and the negative/weak expression of CD45, CD19, and CD27. Patients with higher MFC scores (≥4) were associated with poor survival and ISS stage III and DSS stage III than patients with MFC scores (<4) [20]. In another study, lack of CD27 expression was associated with shortened PFS and OS [21], whereas CD45+ expression had better OS and PFS than CD45- expression [22]; whereas in another study CD45+ expression had inferior OS [23]. In another study conducted by Paiva et al., CD81+ve expression has poor PFS and OS in comparison to CD81-ve expression [24]. The absence of CD200 expression was associated with better PFS [25].

Over the years, advancements in diagnostic and monitoring techniques have led to a deeper understanding of the disease, with a particular focus on MRD. MRD refers to the small number of cancer cells that remain in the body after treatment and cannot be detected by standard diagnostic methods. In the context of MM, MRD assessment involves identifying and quantifying the residual malignant plasma cells in the bone marrow or peripheral blood. The clinical implication of MRD has long been recognised; persistent MRD after treatment indicates that the tumor cells are not completely eradicated, and relapse is expected shortly. Traditional measures of treatment response, such as CR or PR, may not capture the presence of these minimal residual cells, making MRD assessment a valuable tool for a more accurate evaluation of treatment efficacy. Until now, independent studies all show that MRD negativity is a superior prognostic factor in various MM treatment regimens. MRD negativity was positively associated with prolonged PFS and OS in MM.

The induction regimens have a role in the treatment of MM [26], the choice of an induction regimen depends on various factors, including the patient's overall health, the stage of the disease, and the individual treatment plan. In this objective, the regimens used were VRd in 50% of cases followed by VPd in 17% and Vd in 6% of the cases. Although, to determine MRD level, a standard cut-off is ≥0.01% for acute leukemia. However, the value of BM-MRD% for our subset of MM patients was 0.005 (0.002-0.034) which is lower than this cut-off. So, we categorized BM-MRD% into two categories based on the median value of MRD%. BM-MRD positive and BM-MRD negative. BM-MRD% was found to be negative in 66% of patients, while it was found to be positive in 34% of patients.

Correlation and association of BM-MRD% with other clinical parameters revealed no significant correlations between BM-MRD% and Hb, serum albumin, β2-µg, serum creatinine, LDH, BM-PC% by morphology, and BM-PC% at diagnosis. Similarly, no significant associations were found between BM-MRD% and gender, staging, ECOG-PS, FISH, BJ-protein, and response. These results suggest that the level of MRD in the BM does not correlate with these clinical parameters, indicating that BM-MRD% may not be directly influenced by these factors. Notably, the lack of association with FISH suggests that the molecular and cytogenetic characteristics of the disease do not directly impact the BM-MRD% levels.

We analysed the survival outcomes based on MRD status. Our study compared MRD-positive and MRD-negative patients in terms of survival outcomes using BM-MRD measurements. However, there was no significant difference observed between the two groups in terms of survival outcomes. In a recent extensive meta-analysis encompassing data from six randomized trials involving 3,283 newly diagnosed MM patients, it was validated that attaining MRD negativity was significantly associated with an extended PFS [27]. These studies demonstrate the clinical significance and prognostic value of MRD assessment using FCM in MM. MRD negativity assessed by MFC has consistently been shown to correlate with prolonged survival outcomes and is considered an independent predictor of favorable prognosis.

In a study by Rawstron et al., multiparameter flow cytometry (MFC) was used to assess MRD in MM patients and demonstrated that MRD negativity, defined as less than one tumor cell in 10^4 nucleated cells, was associated with significantly prolonged PFS and OS compared to MRD-positive patients [28]. In a retrospective analysis by Flores-Montero et al., MRD assessment by MFC using a 10-color antibody panel showed a high concordance rate of 92.7% with NGS in detecting MRD in MM patients, highlighting the robustness and clinical utility of MFC in MRD evaluation [9]. In a study by Paiva et al., the results demonstrated that MRD negativity assessed by MFC was an independent predictor of improved PFS and OS [5]. Achieving MRD negativity after therapy was associated with significantly prolonged survival outcomes [5]. In a study by Martinez-Lopez et al., MRD evaluation using MFC at different time points during treatment revealed that achieving MRD negativity within the first year of therapy was associated with a significantly longer PFS and OS in MM patients, highlighting the importance of early MRD assessment as a predictive tool for long-term outcomes [29]. In a prospective study by Paiva et al., MRD assessment by MFC at day 100 post-ASCT was shown to be a powerful predictor of clinical outcomes in MM patients, demonstrating the clinical impact of this assessment in a large cohort [30]. MRD negativity at this early time point was associated with significantly improved PFS and OS compared to MRD-positive patients.

These studies demonstrate the clinical significance and prognostic value of MRD at post-induction can provide valuable prognostic information in MM. MRD negativity assessed by MFC has consistently been shown to correlate with prolonged survival outcomes and is considered an independent predictor of favorable prognosis unlike in our study where we could not demonstrate a significant survival advantage. The drawbacks of our study are attrition of cases during follow-up, the smaller sample size for MRD assessment, and the lack of MRD status at day 100 post-ASCT (the most validated time point for MRD assessment).

## Conclusions

In conclusion, this study sheds light on the utility of immunophenotyping as an invaluable diagnostic tool in the management of multiple myeloma (MM) patients. The expression of specific cluster of differentiation (CD) markers in the bone marrow can provide valuable insights into disease characteristics and predict patient outcomes. This suggests a potential way to use these indicators in clinical practice for risk assessment and treatment decision-making. The results of this study could be significant in potentially revising the definitions of high-risk disease and in clinical practice by implementing a risk-adapted initial treatment approach. We compared measurable residual disease (MRD)-positive and MRD-negative patients in terms of survival outcomes. However, no significant difference was observed between the two groups in terms of survival outcomes. Further research and validation studies are warranted to confirm these findings and potentially incorporate these markers into clinical practice for risk stratification and treatment decision-making.
